# Rhythm and time in the premotor cortex

**DOI:** 10.1371/journal.pbio.3000293

**Published:** 2019-06-03

**Authors:** Virginia B. Penhune, Robert J. Zatorre

**Affiliations:** 1 Department of Psychology, Concordia University, Montreal, Quebec, Canada; 2 Laboratory for Brain, Music and Sound Research–BRAMS, Montreal, Quebec, Canada; 3 Montreal Neurological Institute, McGill University, Montreal, Quebec, Canada

## Abstract

Many animals can encode temporal intervals and use them to plan their actions, but only humans can flexibly extract a regular beat from complex patterns, such as musical rhythms. Beat-based timing is hypothesized to rely on the integration of sensory information with temporal information encoded in motor regions such as the medial premotor cortex (MPC), but how beat-based timing might be encoded in neuronal populations is mostly unknown. Gámez and colleagues show that the MPC encodes temporal information via a population code visible as circular trajectories in state space; these patterns may represent precursors to more-complex skills such as beat-based timing.

## Abstract 2

Many animals can encode temporal intervals, but only humans can flexibly extract a regular beat from complex patterns. This Primer explores recent insights into how rhythm and beat are encoded in the brain.

Just listen to Antonio Carlos Jobim’s “Girl from Ipanema” (YouTube version of this song available here). The urge to sway with the languid, syncopated samba rhythm is irresistible. Why does this happen? It somehow must result from interactions between the auditory and motor systems of the brain that detect and anticipate temporally predictable moments in the song. This beat-based form of sensory-motor timing allows humans to flexibly extract a regular temporal structure from a range of rhythms, from simple isochronous sequences, in which all the intervals are identical, to more-complex meters like those of waltzes, marches, and sambas—like Jobim’s piece [1, 2]. People even perceive a regular beat at moments in the music when no sound is present [3], as in the last bar of “Girl,” when the downbeat occurs on a silent rest. Further, motor regions of the brain are active when people listen to musical rhythms, even without moving [4]. These facts demonstrate that the beat is an abstract percept that is not solely dependent on features of the stimulus or motor responses. Beat-based structure is particular to music, but quasiperiodic temporal structure is also present at longer timescales in language [5]. Humans can use temporal predictions based on the beat to facilitate action and perception. Predictable beats enhance attention to stimuli that fall on the beat, resulting in better discrimination or detection [6, 7]. Predictable beats also facilitate movement timing, making it more accurate and less variable [8]. This may be the reason why athletes in many sports use music to guide movement timing.

Beat-based timing can be contrasted with interval timing—the ability to encode and remember the interval between two events [9]. Many animals can encode temporal intervals and use them to plan their actions [10]. Your dog knows when it is time for dinner and how long it takes to pour the food. In contrast, beat-based timing appears to be characteristically human. Beat-based timing in humans has been well characterized using a deceptively simple paradigm known as the tapping and continuation task (see [11] for review). A series of equally spaced stimuli are presented, and people synchronize their finger taps to them. Then the stimuli are stopped, and participants continue to tap, maintaining the original pace. Many variables can be manipulated in this task, including stimulus modality, tempo, regularity of timing, etc. The measures extracted from it are period and phase accuracy, as well as variability. Across a large number of experiments, this simple task reveals several important features of human rhythmic timing: (1) it is predictive, with taps preceding the onset of stimuli after only a few cycles; (2) it is more accurate for auditory than visual stimuli; (3) accuracy declines linearly for longer intervals; and (4) people can maintain the correct pace for many cycles after the stimuli cease.

Because beat-based timing in humans favors auditory stimuli, it has been hypothesized that this ability is related to neural specialization for audio-motor coupling required for language [12]. Neuroimaging studies in humans have identified a network of auditory and motor structures involved in both interval- and beat-based timing [12–14]. These include posterior auditory regions, inferior parietal, premotor, and supplementary motor areas (SMAs/preSMAs in humans, medial premotor cortex [MPC] in monkeys [15]). Subcortical structures also contribute, with basal ganglia thought to be more important for beat-based timing and the cerebellum for interval timing [16, 17]. A number of influential models suggest that motor planning regions such as the SMA/MPC may be critical in encoding temporal intervals and providing this information to sensory regions to guide perception [18–20]. The neural mechanism underlying beat-based timing is proposed to be nested oscillations in auditory and motor regions that underlie a dynamic interaction between these systems [21–24]. But until now, how these oscillatory mechanisms are actually instantiated in neuronal populations has never been satisfactorily described.

Since interval timing is common in animals, whereas beat-based timing appears to be unique to humans, there has been much interest in identifying whether nonhuman primates possess related abilities that might be considered precursors of these processes. Identifying precursors would allow us to causally test the contributions of specific brain regions and to model encoding mechanisms at the neuronal level in a way that is difficult to do in humans. To examine possible precursor abilities and their underlying mechanisms in the macaque, previous studies have developed a monkey paradigm based on the tapping and continuation task in humans [25]. Like the human task, monkeys tap in synchrony with a series of stimuli and continue after they stop. Unlike the human task, the stimuli are typically visual, because monkeys have difficulty engaging with auditory stimuli, and the sequences are quite short: only 3 taps compared to the usual 30 in humans. Using this innovative paradigm, it has been shown that macaques can perform this task across a range of tempos and that their responses can show some degree of predictive timing if enough behavioral feedback is given [26]. This observation suggests that macaques may possess an intermediate form of event timing that allows them to make temporal predictions from a series of simple intervals. This form of timing may be similar to the countdown in a game of tag or a rocket launch, in which a regularly spaced series of cues allows us to accurately predict when to run or start the engines.

In the target paper, Gámez and colleagues [27] trained two macaques to perform this tapping and continuation task while recording neurophysiological activity simultaneously from a large number of neurons within the MPC. Behaviorally, the monkeys learned to tap to a rhythmic visual cue at different speeds and to continue tapping after the stimulus stopped. The neural data were analyzed using principal component analyses (PCAs), a multivariate approach that converts the data into a representation in which the variance is accounted for by a series of uncorrelated (orthogonal) components. This analytical approach is able to reveal organizational features of complex data that would not be identified by simple linear or univariate approaches (such as spike averaging).

The most remarkable finding is that as the monkeys tap, the neural responses, modeled in a multidimensional space defined by the PCA, describe a circular trajectory of constant speed whose radius scales with the duration of the time intervals (i.e., the tempo of the sequence) (see the Supporting Information for a video illustrating this phenomenon). The relation to tempo indicates that the circular trajectory encodes relevant properties of the temporal sequence. These trajectories are found not only when the animal is tapping in synchrony to the stimulus but also during the continuation phase when there is no stimulus. Therefore, the brain activity is not merely a response to a stimulus. Furthermore, one monkey performed a control task using stimulus sequences that were unpredictable in time; under those conditions, the monkey was slower to respond, and also the neural trajectories were much less well organized. Thus, the shape of the neural trajectories is related to the degree of temporal regularity. Finally, these neural population state trajectories may be related to previously reported single-cell ramping activity during performance of this task [28].

One might ask if the brain activity is directly related to the movements themselves: analysis of the kinematics of the monkey’s finger shows that the movement trajectory is not sinusoidal, as one might expect if it followed the circular trajectory directly. Conversely, the periodicity of the neural activity is closely related to the onset of movement, so it appears that when the neural trajectory reaches a certain position in state space, there is a triggering of the motor action. Finally, the authors show that the results are stable even when data from fewer neurons are analyzed, indicating that the results are robust and correspond to a distributed, sparse population code throughout the premotor region.

This set of findings provides remarkable insight into the mechanisms by which entrainment is instantiated in the MPC, a key cortical region for this ability. The regularity of the circular neural trajectories, their continuation even in the absence of a stimulus, and the relationship between their features and behavior (sensitivity to tempo and to temporal structure) strongly suggest that they represent an internal sensorimotor representation of the timed sequence. A key advance in this paper is the idea that timing is encoded in a neural population clock—a mechanism that may be unique to the MPC or that may be a common code in other regions involved in timing. As with any important new finding, this one raises questions and opens many possibilities for future research.

Coming back to the distinction between interval-based and beat-based timing, one might ask which one is pertinent to the proposed neural mechanism in the MPC. Although the monkey findings point to a certain level of abstractness in the internal representation (the pattern can encode any tempo and is independent of the presence of the stimulus itself), it is nonetheless unlikely to represent a true beat-based timing mechanism, since monkeys seem to lack this ability. Rather, it likely pertains to interval-based timing, albeit a more complex version of it, involved in entrainment to a sequence of isochronous events [29].

But could the mechanism uncovered here represent a precursor to the more complex beat-processing ability that humans express so readily? It’s hard to say, because if the two mechanisms rely on distinct neural systems in humans, as has been proposed, then the mechanism uncovered in monkeys would pertain only to the interval-based aspect. However, it seems likely that a beat-based mechanism could only exist if regular, timed intervals are well represented in the first place, so in that sense, the findings here may represent a precursor upon which the human nervous system has built more-complex systems. The question then becomes, what are those more complex systems, exactly? Is it simply a matter of quantitative enhancement of what already exists in a monkey? It is notable, for example, that although the monkeys are clearly entraining, they do so more sluggishly than humans, and they follow the stimulus rather than align with it or predict it as humans tend to do. So perhaps all you need is greater precision. Or instead, there may well be a qualitatively different system that allows beat-based timing but that interacts with interval-based mechanisms.

One valuable aspect of these findings is that they provide a concrete and sophisticated model that can be tested in humans. Although we cannot easily perform multiunit recordings in humans (except perhaps with intracranial techniques in epilepsy patients, for instance), we can think of ways to use neuroimaging methods to test the model. For instance, with high-field functional MRI, duration-related topographies have already been demonstrated in the SMA [30]. Therefore, it may also be possible to image sufficiently small voxels and apply PCA (or other multivariate approaches) to test for the existence of neural trajectories in SMA similar to those seen in monkeys. The temporal resolution needed may be an issue for MRI, but techniques such as magnetoencephalography could perhaps be applied in a similar vein. These approaches might help us to understand whether interval- and beat-based timing are truly dissociable or on a continuum.

Neuroimaging approaches applied to this issue would have the further advantage that they would help reveal more information about the network in which the MPC is embedded, because the entire brain is typically imaged simultaneously. Gámez and colleagues point out that the MPC is part of both the cortico-basal ganglia and the cortico-cerebellar circuits; it may therefore participate in both interval and rhythmic timing [16, 17]. Nonetheless, the neurophysiological findings do not tell us whether the patterns observed represent an intrinsic mechanism of the MPC or reflect readout from upstream regions—for example, frontal areas. We need to understand what the relevant inputs are in order to better understand how sensory and motor information are integrated at a network level. Similarly, it is not clear how the outputs from the MPC to downstream motor structures—such as dorsal premotor cortex, primary motor cortex, basal ganglia, and cerebellum—lead to the organization of action that enables precise, timed movements on the beat. Most critically, we need to understand what computations are carried out across a distributed network of regions, such that sensory and motor system activity becomes part of an integrated whole. Such knowledge would indirectly help to answer the evolutionary question of whether human musical and timing skills depend on similar, if enhanced, mechanisms as in monkeys or engage additional, qualitatively distinct mechanisms within more-complex networks of neural structures. Monkeys may never swing to Brazilian samba, but their movements may nonetheless help us to figure out why we do.

## Supporting information

S1 Video Medial premotor cortex neural population trajectories during the continuation condition of the tapping task. The trajectory completes an oscillatory cycle on every produced interval, with an amplitude that is larger for the slower tempo (red) compared to the faster tempo (blue). The tapping times for the two tempos are displayed at the bottom.
(MP4)

## Abbreviations

MPC: medial premotor cortex
PCA: principal component analysis
SMA: supplementary motor area
